# Supplementary material for: Methodological review to develop a list of bias items for adaptive clinical trials: Protocol and rationale
Source: PLoS One. 2024 Dec 12;19(12):e0303315. doi: 10.1371/journal.pone.0303315 (PMC11637403; doi:10.1371/journal.pone.0303315)
Supplement: S1 File — (DOCX) [file pone.0303315.s001.docx]

**S1 File.** Search strategies

| **Study types 1 and 2**  **Ovid MEDLINE (R) and Epub Ahead of Print, In-Process & Other Non-Indexed Citations, Daily and Versions(R) (1946 - ):**  ((Adaptive Clinical Trial* or Adaptive Design*) adj5 (tool? or instrument? or checklist? or check list? or scale? or measure? or assess? or compar*)).ab,ti.  **Ovid EMBASE (R) and Epub Ahead of Print, In-Process & Other Non-Indexed Citations, Daily and Versions(R) (1946 - ):**  ((Adaptive Clinical Trial* or Adaptive Design*) adj5 (tool? or instrument? or checklist? or check list? or scale? or measure? or assess? or compar*)).ab,ti.  **Web of Science (R) and Epub Ahead of Print, In-Process & Other Non-Indexed Citations, Daily and Versions(R) (1946 - ):**  ((Adaptive Clinical Trial* or Adaptive Design*) adj5 (tool? or instrument? or checklist? or check list? or scale? or measure? or assess? or compar*)).ab,ti. |
| --- |

**EBM Reviews -** **Cochrane Central Register of Controlled Trials (CENTRAL):**

((Adaptive Clinical Trial* or Adaptive Design*) adj5 (tool? or instrument? or checklist? or check list? or scale? or measure? or assess? or compar* or valid$ or invalid or bias$ or apprais$ or quality)).ab,ti.

**EBM Reviews - Cochrane Methodology Register (includes Cochrane Colloquium abstracts):**

((Adaptive Clinical Trial* or Adaptive Design*) adj5 (tool? or instrument? or checklist? or check list? or scale? or measure? or assess? or compar* or valid$ or invalid or bias$ or apprais$ or quality)).ab,ti.

**EBM Reviews - Cochrane Library:**

((Adaptive Clinical Trial* OR Adaptive Design*) AND (tool? or instrument? or checklist? or check list? or scale? or measure? or assess? or compar* or valid$ or invalid or bias$ or apprais$ or quality)):ti,ab,kw

**The EQUATOR Network:**

http://www.equator-network.org/reportingguidelines/) Free text search: “adaptive trials”

**ProQuest Dissertations & Theses Global**:

TI(adaptive clinical trials) or TI(adaptive trial) and AB(tool)

**Cochrane Comparing Multiple Interventions Methods Group:** https://methods.cochrane.org/cmi/welcome https://methods.cochrane.org/cmi/relevant-publications-and-resources

**Scientific Resource Center Methods library of the AHRQ Effective Health Care Program:** http://www.refworks.com/refworks2/?site=027181135918800000%2F57381342557464357%2FSRC+Methods+ Library “adaptive clinical trials”, “adaptive design”, “adaptive trials”, “Bayesian trials”, “Bayesian clinical trials”, “platform”, “multi-arm, multi-stage”

**International Network of Agencies for Health Technology Assessment:**

https://www.inahta.org “adaptive clinical trials”, “adaptive design”, “adaptive trials”, “Bayesian trials”, “Bayesian clinical trials”, “platform”, “multi-arm, multi-stage”

**Pharmaceutical Benefits Advisory Committee:** https://www.pbs.gov.au/info/industry/listing/participants/pbac “adaptive clinical trials”, “adaptive design”, “adaptive trials”, “Bayesian trials”, “Bayesian clinical trials”, “platform”, “multi-arm, multi-stage”

**Institut für Qualität und Wirtschaftlichkeit im Gesundheitswesen:** https://www.iqwig.de/en/home.2724.html “adaptive clinical trials”, “adaptive design”, “adaptive trials”, “Bayesian trials”, “Bayesian clinical trials”, “platform”, “multi-arm, multi-stage”

**European Network for Health Technology Assessment:**

https://eunethta.eu/methodology-guidelines/ “adaptive clinical trials”, “adaptive design”, “adaptive trials”, “Bayesian trials”, “Bayesian clinical trials”, “platform”, “multi-arm, multi-stage”

**Guidelines International Network:**

https://g-i-n.net/home “adaptive clinical trials”, “adaptive design”, “adaptive trials”, “Bayesian trials”, “Bayesian clinical trials”, “platform”, “multi-arm, multi-stage”

**International Society for Pharmacoeconomics and Outcomes Research:**

https://www.ispor.org/ AND https://tools.ispor.org/peguidelines “adaptive clinical trials”, “adaptive design”, “adaptive trials”, “Bayesian trials”, “Bayesian clinical trials”, “platform”, “multi-arm, multi-stage”

**National Institute for Health and Care Excellence Decision Support Unit:** http://nicedsu.org.uk/multivariate-meta-analysis-tsd “adaptive clinical trials”, “adaptive design”, “adaptive trials”, “Bayesian trials”, “Bayesian clinical trials”, “platform”, “multi-arm, multi-stage”

**Canadian Agency for Drugs and Technologies in Health:**

https://www.cadth.ca/ Search study type “reports”, then for “adaptive clinical trials”, “adaptive design”, “adaptive trials”, “Bayesian trials”, “Bayesian clinical trials”, “platform”, “multi-arm, multi-stage”

**WHO International Clinical Trials Registry Platform (ICTRP):**

https://trialsearch.who.int/ “adaptive clinical trials”, “adaptive design”, “adaptive trials”, “Bayesian trials”, “Bayesian design”, “Bayesian clinical trials”, “platform”, “multi-arm, multi-stage”

**NIH Clinical Trials Database:**

https://clinicaltrials.gov “adaptive clinical trials”, “adaptive design”, “adaptive trials”, “Bayesian trials”, “Bayesian design”, “Bayesian clinical trials”, “platform”, “multi-arm, multi-stage”
