# Supplementary material for: Methodological review to develop a list of bias items for adaptive clinical trials: Protocol and rationale
Source: PLoS One. 2024 Dec 12;19(12):e0303315. doi: 10.1371/journal.pone.0303315 (PMC11637403; doi:10.1371/journal.pone.0303315)
Supplement: S2 File — (DOCX) [file pone.0303315.s002.docx]

**S2 File.** PRISMA flow diagram for prospective article screening and full-text review.

**Identification of new studies or tools via other methods**

**Identification of new studies or tools via databases and registers**

Records identified from:

Databases

Registers

Records removed *before screening*:

Duplicate records removed

Records marked as ineligible by automation tools

Records removed for other reasons

Records identified from:

Websites

Organisations

Grey literature databases

Citation searching

**Identification**

Total studies included in review

Reports of total included studies

Reports assessed for eligibility

Reports sought for retrieval

Records screened

Records excluded

Reports not retrieved

Reports sought for retrieval

Reports not retrieved

**Screening**

Reports excluded:

Reports excluded:

Reports assessed for eligibility

New studies included in review

Reports of new included studies

**Included**
