# Supplementary material for: Methodological review to develop a list of bias items for adaptive clinical trials: Protocol and rationale
Source: PLoS One. 2024 Dec 12;19(12):e0303315. doi: 10.1371/journal.pone.0303315 (PMC11637403; doi:10.1371/journal.pone.0303315)
Supplement: Attachment — Submitted filename: PONE-D-24-13528 - Response to reviewers.docx [file pone.0303315.s004.docx]

PONE-D-24-13528
Methodological review to develop a list of bias items for adaptive clinical trials: Protocol and rationale
PLOS ONE

Dear Dr. Staibano,

Thank you for submitting your manuscript to PLOS ONE. After careful consideration, we feel that it has merit but does not fully meet PLOS ONE’s publication criteria as it currently stands. Therefore, we invite you to submit a revised version of the manuscript that addresses the points raised during the review process.

Please submit your revised manuscript by Aug 17 2024 11:59PM. If you will need more time than this to complete your revisions, please reply to this message or contact the journal office at [plosone@plos.org](mailto:plosone@plos.org). When you&apos;re ready to submit your revision, log on to <https://www.editorialmanager.com/pone/> and select the &apos;Submissions Needing Revision&apos; folder to locate your manuscript file.

Please include the following items when submitting your revised manuscript:

- A rebuttal letter that responds to each point raised by the academic editor and reviewer(s). You should upload this letter as a separate file labeled &apos;Response to Reviewers&apos;.
- A marked-up copy of your manuscript that highlights changes made to the original version. You should upload this as a separate file labeled &apos;Revised Manuscript with Track Changes&apos;.
- An unmarked version of your revised paper without tracked changes. You should upload this as a separate file labeled &apos;Manuscript&apos;.

If you would like to make changes to your financial disclosure, please include your updated statement in your cover letter. Guidelines for resubmitting your figure files are available below the reviewer comments at the end of this letter.

If applicable, we recommend that you deposit your laboratory protocols in <protocols.io> to enhance the reproducibility of your results. <Protocols.io> assigns your protocol its own identifier (DOI) so that it can be cited independently in the future. For instructions see: <https://journals.plos.org/plosone/s/submission-guidelines#loc-laboratory-protocols>. Additionally, PLOS ONE offers an option for publishing peer-reviewed Lab Protocol articles, which describe protocols hosted on <protocols.io>. Read more information on sharing protocols at <https://plos.org/protocols?utm_medium=editorial-email&utm_source=authorletters&utm_campaign=protocols>.

We look forward to receiving your revised manuscript.

Kind regards,

Sathish Muthu
Academic Editor
PLOS ONE

Journal requirements:

When submitting your revision, we need you to address these additional requirements.

1. Please ensure that your manuscript meets PLOS ONE&apos;s style requirements, including those for file naming. The PLOS ONE style templates can be found at

<https://journals.plos.org/plosone/s/file?id=wjVg/PLOSOne_formatting_sample_main_body.pdf> and

<https://journals.plos.org/plosone/s/file?id=ba62/PLOSOne_formatting_sample_title_authors_affiliations.pdf>

2. When completing the data availability statement of the submission form, you indicated that you will make your data available on acceptance. We strongly recommend all authors decide on a data sharing plan before acceptance, as the process can be lengthy and hold up publication timelines. Please note that, though access restrictions are acceptable now, your entire data will need to be made freely accessible if your manuscript is accepted for publication. This policy applies to all data except where public deposition would breach compliance with the protocol approved by your research ethics board. If you are unable to adhere to our open data policy, please kindly revise your statement to explain your reasoning and we will seek the editor&apos;s input on an exemption. Please be assured that, once you have provided your new statement, the assessment of your exemption will not hold up the peer review process.

3. Please include captions for your Supporting Information files at the end of your manuscript, and update any in-text citations to match accordingly. Please see our Supporting Information guidelines for more information: <http://journals.plos.org/plosone/s/supporting-information>.

4. Please review your reference list to ensure that it is complete and correct. If you have cited papers that have been retracted, please include the rationale for doing so in the manuscript text, or remove these references and replace them with relevant current references. Any changes to the reference list should be mentioned in the rebuttal letter that accompanies your revised manuscript. If you need to cite a retracted article, indicate the article’s retracted status in the References list and also include a citation and full reference for the retraction notice.

Additional Editor Comments (if provided):

**I appreciate the effort put forth in this work and the importance of developing a risk-of-bias tool for adaptive clinical trials (ACTs). This is a crucial step towards improving the quality and transparency of ACTs, which have the potential to streamline clinical research. I would the authors to provide a clear view of the Adaptive clinical trials for the benefit of the authors in the introduction with examples and their advantages and limitations. Further in the methodological aspect does the authors consider the tool as a standalone tool or as a additive tool to RoB2 is not clearly made out. Analysis need to be detailed on the type of regression to be utilized.**

We have removed the regression analysis, as we do not feel as though it contributes to development of a risk of bias tool for ACTs. We stated in the methods section that we will plan to create a standalone tool, but as the process enters the Delphi and candidate tool development stages, we will determine the role of instead creating an extension to the Cochrane RoB 2.0 tool.

**Comments to the Author**

1. Does the manuscript provide a valid rationale for the proposed study, with clearly identified and justified research questions?

The research question outlined is expected to address a valid academic problem or topic and contribute to the base of knowledge in the field.

Reviewer #1: Yes

2. Is the protocol technically sound and planned in a manner that will lead to a meaningful outcome and allow testing the stated hypotheses?

The manuscript should describe the methods in sufficient detail to prevent undisclosed flexibility in the experimental procedure or analysis pipeline, including sufficient outcome-neutral conditions (e.g. necessary controls, absence of floor or ceiling effects) to test the proposed hypotheses and a statistical power analysis where applicable. As there may be aspects of the methodology and analysis which can only be refined once the work is undertaken, authors should outline potential assumptions and explicitly describe what aspects of the proposed analyses, if any, are exploratory.

Reviewer #1: Yes

3. Is the methodology feasible and described in sufficient detail to allow the work to be replicable?

Descriptions of methods and materials in the protocol should be reported in sufficient detail for another researcher to reproduce all experiments and analyses. The protocol should describe the appropriate controls, sample size calculations, and replication needed to ensure that the data are robust and reproducible.

Reviewer #1: Yes

4. Have the authors described where all data underlying the findings will be made available when the study is complete?

The [PLOS Data policy](https://journals.plos.org/plosone/s/materials-and-software-sharing) requires authors to make all data underlying the findings described in their manuscript fully available without restriction, with rare exception, at the time of publication. The data should be provided as part of the manuscript or its supporting information, or deposited to a public repository. For example, in addition to summary statistics, the data points behind means, medians and variance measures should be available. If there are restrictions on publicly sharing data—e.g. participant privacy or use of data from a third party—those must be specified.

Reviewer #1: No

5. Is the manuscript presented in an intelligible fashion and written in standard English?

PLOS ONE does not copyedit accepted manuscripts, so the language in submitted articles must be clear, correct, and unambiguous. Any typographical or grammatical errors should be corrected at revision, so please note any specific errors here.

Reviewer #1: Yes

6. Review Comments to the Author

Please use the space provided to explain your answers to the questions above and, if applicable, provide comments about issues authors must address before this protocol can be accepted for publication. You may also include additional comments for the author, including concerns about research or publication ethics.

You may also provide optional suggestions and comments to authors that they might find helpful in planning their study.

(Please upload your review as an attachment if it exceeds 20,000 characters)

**Reviewer #1: Thank you for the opportunity to review your manuscript entitled "Methodological review to develop a list of bias items for adaptive clinical trials: Protocol and rationale." I appreciate the effort put forth in this work and the importance of developing a risk-of-bias tool for adaptive clinical trials (ACTs). This is a crucial step towards improving the quality and transparency of ACTs, which have the potential to streamline clinical research. I hope the following comments and suggestions will improve the quality of the manuscript.**

**Abstract
1. The authors mentioned that "we will perform regression analysis to identify factors associated with poor reporting quality and high risk of bias," but this was not discussed in the methods section in the main text. Please specify the type of regression analysis to be used (e.g., Poisson, linear, logistic) and provide details on the variables to be included in the regression model in the main text.**

Thank you. This was an oversight and was not removed from a previous version of the draft. We do not believe that a regression analysis will contribute to the development of a risk of bias tool.

**2. It would be helpful to provide more details on the planned dissemination strategy (e.g., publication, conference presentation, or any other strategies) for the developed risk-of-bias tool, beyond mentioning that this is the first step in its development.**

We have added to this section.

**Introduction
3. The description of Adaptive clinical trials (ACTs) does not provide a clear picture of the concept. Please consider explaining the concept in this section to facilitate better understanding of the later sections.**

Thank you. We further explained this concept.

**4. Adaptive clinical trials (ACTs) is mentioned twice in one sentence: "Adaptive clinical trials (ACTs) Adaptive clinical trial (ACTs)." Please revise for better clarity.**

This has been amended. Thank you.

**5. The authors may consider briefly mentioning the potential challenges or limitations of ACTs, in addition to the advantages, to further highlight the importance of a risk-of-bias tool.**

We have further added to the limitations of ACTs. Thank you.

**Methods
6. Please consider providing a brief overview or example of the Delphi process and subsequent steps planned for the development and validation of the risk-of-bias tool.**

This has now been included. Thanks.

**7. It is unclear how prospective/retrospective cohort, cross-sectional, and case series studies could provide information related to methodological bias, reporting, or quality in ACTs. Please clarify or revise the inclusion criteria under: "Study type 2: Studies that describe items relating to methodological bias, reporting, or quality in ACTs."**

We agree. Those study types will realistically not evaluate methodological quality in ACTs. We have thus changed this to only include study types that are likely to include this information. Further, we have amended this to only include two study types (1) studies that have described or suggested any risk of bias or methodological quality items for ACTs and (2) any existing studies that have evaluated methodological bias/quality in ACTs. We have removed those study types. We will also not be looking for any published ACTs since this will not contribute to the identification of risk of bias items.

**8. The authors mentioned "Eligibility: There will be three types of studies included in this scoping review." Did the authors mean methodological review instead of scoping review?**

Yes, this was changed. Thank you.

**9. Please clarify whether the authors plan to include studies that assess the methodological quality or risk of bias of ACTs using tools designed for traditional RCTs (e.g., Cochrane RoB 2.0, Jadad scale), or if the focus is solely on tools specifically designed for ACTs.**

We will include those tools as well. We have stated this more clearly.

**Discussion
10. Please expand on the potential implications and impact of developing a risk-of-bias tool for ACTs, both for researchers and clinicians.**

We have done this. Thank you.

7. PLOS authors have the option to publish the peer review history of their article ([what does this mean?](https://journals.plos.org/plosone/s/editorial-and-peer-review-process#loc-peer-review-history)). If published, this will include your full peer review and any attached files.

If you choose “no”, your identity will remain anonymous but your review may still be made public.

**Do you want your identity to be public for this peer review?** For information about this choice, including consent withdrawal, please see our [Privacy Policy](https://www.plos.org/privacy-policy).

Reviewer #1: No
